# The Maximum Growth Temperature for Eukaryotes Is Thermodynamically Driven but Ecologically Contingent

**DOI:** 10.3390/life16061016

**Published:** 2026-06-17

**Authors:** William Bains

**Affiliations:** School of Physics & Astronomy, Cardiff University, 4 The Parade, Cardiff CF24 3AA, UK; bainsw@cardiff.ac.uk

**Keywords:** eukaryote, growth temperature, thermophily, maximum temperature, intrinsically disordered protein, genome, fungi, oomycete

## Abstract

Temperature is a state variable that affects all life. While it is known that archaea can grow at 120 °C and many bacteria can grow at over 100 °C, no eukaryote is known to complete a life cycle at above 65 °C. This paper explores why the difference in the maximum temperature of eukaryotes and other kingdoms of life might occur. It finds that chemical and genome structural differences between the domains of life are unlikely to explain the difference in maximum growth temperature, with the exception of the Saccharomycotina, which are different from other fungi, possibly because of their unique ecology. The distribution of inherently disordered proteins (IDPs), however, is significantly correlated with maximum and minimum growth temperature in fungi, and with the range of temperatures over which fungi can grow. I also demonstrate that the range of temperatures over which a species can grow is correlated with its maximum temperature. I postulate that the range is correlated with maximum temperature because all real-world ecologies fluctuate between elevated and average surface temperatures, and the thermodynamics of IDP-based structures in eukaryotes inherently limits the range over which they can operate. Thus, the 65 °C maximum temperature limit for eukaryotes is a result of a combination of thermodynamic properties of their organization and the temperature regime on the modern Earth; I suggest an experimental approach to testing this.

## 1. Introduction

Temperature is a key thermodynamic variable that influences every process in a living organism [1]. For most organisms, temperature is a state variable to which they must adapt, and only large organisms with active metabolisms can maintain an internal temperature substantially different from that of their environment. Most organisms adapt to high or low temperature by a wide range of metabolic adaptations that allow them to function at the temperature that is ambient in their usual environment (reviewed in [2,3,4]).

Organisms are known to grow at 120 °C [5,6,7], and in situ characterization of organisms in hydrothermal vents [8] and analysis of the thermal stability of metabolites [9] hint at active life up to 150 °C. However, no eukaryote is known to survive above 65 °C as actively growing organisms ([1,10,11,12]) (claims of metazoans able to live at temperatures of 80 °C are probably incorrect [13]). Many can survive in sporulated, encysted, or ‘tun’ forms at much higher temperatures, but a stable ecosystem cannot be built of such inert organisms [1].

Understanding the limitations that apply to complex life is of fundamental interest in predicting where in the universe such life might occur [14]. It is also of practical importance for predicting how potentially infectious agents such as fungi and protozoa could adapt to changing ecosystem temperatures, and thereby potentially become new infectious agents in mammals and birds [15,16,17,18,19,20], and for industrial processes [21,22].

Many of the biochemical adaptations that allow organisms to grow above 100 °C (reviewed in [23,24,25]) are, in principle, applicable to eukaryotic biochemistry. The chemistry of archaea and bacteria (here referred to as ‘prokaryotes’ for convenience) on the one hand, and eukaryotes on the other, is very similar. The chemical and protein structural adaptations to hyperthermophily seen in prokaryotes (see e.g., [23,26,27,28,29,30,31,32,33]) could, in principle, be seen in eukaryotes, and indeed, in some cases, are seen (e.g., [34,35,36]). Adaptations such as changes in membrane fluidity are common in eukaryotes [34,35,37]; failures of specific organisms to survive above mesophilic temperatures (e.g., [38]) only show that a specific organism cannot adapt indefinitely over a single life cycle, not that the clade as a whole could not evolve broader temperature tolerance.

A range of specific features of eukaryotic biology have been suggested as limits to eukaryotic thermophily, such as genome size and composition, membrane composition [39,40,41,42,43,44], and the ability of membranes to form organelles [45,46]. However, there is limited support for any of these explanations.

One rarely discussed issue is the ‘maintenance energy’ of the cell, the basic energy requirement for maintaining the cell’s components in a functional state, ready to grow or divide. Maintenance energy increases exponentially with temperature [47]. Maintenance energy is higher for aerobic than anaerobic growth [48], but despite their greater complexity and (generally) larger cell size, there is no difference between the maintenance energy for prokaryotes and eukaryotes expressed as watts/gram biomass [49]. If the hypothesis that the primary difference between eukaryotes and prokaryotes is the former’s ability to capture more energy per gram biomass [50,51], then we might expect eukaryotes to be better adapted to the high energy demands of hyperthermophily than are prokaryotes. That this is demonstrably not so suggests that available metabolic energy is not a limiting factor.

One obvious reason for the lack of hyperthermophilic eukaryotes is size. There are few extensive environments on Earth that are constantly at >50 °C. A hot spring pool with a volume of a few cubic meters can only maintain a self-contained ecosystem of small organisms. Thus, we would not expect hyperthermophilic equivalents of elephants or trees. However, unicellular eukaryotes, and in particular yeasts, which have cell sizes comparable to larger bacteria, could colonize such environments.

If core biochemical differences between prokaryotes’ and eukaryotes’ ability to adapt to high temperatures are not in chemical composition, energetics or size, then they must lie elsewhere. The most obvious difference between prokaryotes and eukaryotes is the role of structural order in the control of cellular processes, both through internal partitioning of the cells and the role of structural features such as phase-change organelles in eukaryotes. While these are known in prokaryotes, they dominate eukaryotic control processes. Much of this phase change phenomenon is controlled or driven by intrinsically disordered proteins.

### 1.1. Intrinsically Disordered Proteins

Intrinsically disordered proteins (IDPs) are proteins in which substantial regions of the protein do not form an ordered structure in isolation, either in solution or in a crystal [52]. Rather, the intrinsically disordered region forms a highly variable, semi-random structure in isolation. This may form a more defined structure in cooperation with other macromolecules or may remain disordered in interaction with itself or other molecules to form membrane-less phase-changed compartments within the cell (‘biomolecular condensates’; see [53,54,55,56,57]). While IDPs are known in bacteria and archaea [58,59], they are more prevalent and are believed to be more central actors in cellular structure and control in eukaryotes [59,60]. Roles include chromatin structure and gene control [61,62], larger scale nuclear structure [63], nuclear pore function [64], formation of peroxisomes [65], local enzyme activity [66,67], and movement of structures within the cell [68], and they may include catalysing redox chemistry [69].

This dominant role of structure in control systems, and specifically the role of IDPs, in eukaryotes is expected to have thermodynamic consequences. Structural changes are inherently changes in order, which have associated changes in entropy that make them sensitive to temperature. IDPs are therefore of potential relevance to the temperature range of eukaryotes and are a focus of this study.

### 1.2. Fungi as a Test Group

**Figure 1:**
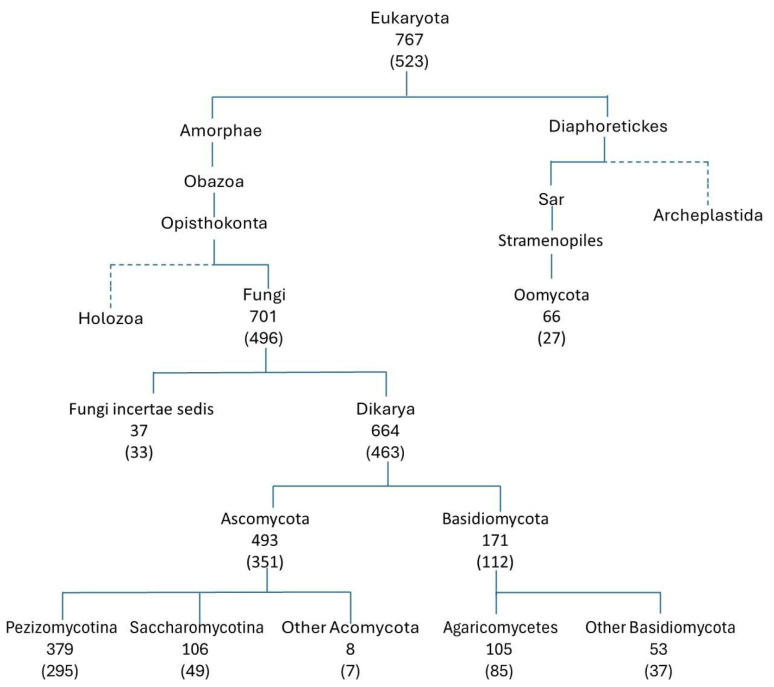
Taxonomy of organisms used in this study. A sketch taxonomic tree shows the broad classification of organisms in this study. Numbers below each class are the number of genomes, numbers in brackets are the numbers of proteomes. Not all taxonomic levels are shown here, and fungal groups in the ascomycetes and basidiomycetes that have few genome sequences are grouped in the diagram, as they are in the analysis below. Dashed lines indicate where the holozoa (which include the animals) and archeplastida (which include the plants) are placed in the taxonomy.

This paper seeks to formulate a hypothesis about the degree to which genome, proteome, and IDP statistics might give insight into why eukaryotes are limited to 65 °C when the chemically very similar archaea can grow at up to 120 °C. I chose the fungi as a test group. Fungi are a large, diverse, monophyletic group of complex eukaryotes that can be conveniently grown in the laboratory [70,71]. The growth temperature of plants (as well as cyanobacteria) is limited by the thermal stability of chlorophyll [72,73]. Animal survival is complicated by the relationship among activity, development, survival, and temperature. Fungi can be cultured for their entire growth cycle in the laboratory, and they show a range of optimal growth temperatures from below 15 °C to 60 °C [74,75,76,77]. They are therefore a test case in which the relationship between physiological features and growth temperature may be explored. A small number of oomycetes were also analysed (see Section 2.1). Fungi and oomycetes have similar life cycles and morphologies and are saprophytic eukaryotes. However, they are evolutionarily distinct, with oomycetes being more closely related to plants than to the opisthokonts (fungi and animals; see Figure 1) [78,79]. Inclusion of oomycetes is therefore a control to test whether findings are specific to fungi.

### 1.3. Structure of This Study

This study examines whether genome, proteome, and IDP statistics are correlated with growth temperatures in fungi and oomycetes, with the aim of developing a hypothesis explaining the apparent temperature limit of 65 °C for eukaryotes, thereby suggesting further observations or experiments that might explain that limit. I start with a summary of genome statistics, which I find to have limited predictive potential, although the fraction of the genome that codes for protein is weakly correlated with growth temperatures. I then explore protein statistics, the nature and distribution of IDPs in organisms of different temperatures, and their correlations with growth temperatures. This provides correlative evidence for the hypothesis that adaptation to high temperatures may require more control agility than mesophilic temperatures. I suggest that this is the result of the need to adapt to a range of temperatures, not an absolute maximum, and provide initial evidence for this. I conclude with suggested tests for this hypothesis.

## 2. Databases and Methods

### 2.1. Genome, Proteome, and Growth Temperature Data

Genome and proteome data, along with growth temperatures, were identified for 701 fungal species and 66 oomycete species through a literature search. For a detailed description of the methods, see [80]. In brief, the literature was searched using Google Scholar for data on the growth temperatures of fungal and oomycete species, and when such data was found, genome and proteome data for that species was searched for; literature referencing growth temperature was then manually searched for references and citations to identify additional data. The dataset reported in [80] has been augmented for this study with an additional 35 oomycetes and 2 new fungi, using the same search strategies. The numbers and classifications of species are summarized in Figure 1. Fungi are grouped into classes with a substantial fraction of the dataset in them.

### 2.2. Intrinsic Disorder Prediction

Intrinsic disorder was predicted using the IUPred3 algorithm [81,82], implemented in Python (version 3.10.11). Proteins from proteome files were individually analysed, the intrinsic disorder per amino acid calculated, and whole-proteome summary statistics compiled, as described in the Results section. The full set of fungal species, genome and proteome statistics and Intrinsically Disordered Protein prediction statistics are available as supplementary information to this paper.

### 2.3. Bacterial Motility Survey

Abstracts of 10,406 papers from the International Journal of Systematic and Evolutionary Microbiology were downloaded from Web of Science (accessed under license to Cardiff University, 22 August 2025), and the text was analysed for keywords relating to temperature, motility, or non-motility. A total of 2629 species report abstracts contained unambiguous identification of growth temperature and motility status, of which 1215 gave a range of temperatures. Of these, 97.3% of abstracts referred to bacteria, and 2.7% referred to archaea.

### 2.4. Statistical Methods

All calculations and graphing were performed in Excel (Microsoft 365 MSO Version 2408 Build 16.0.17928.20114). Principal component analysis was performed using the Real Statistics Resource Pack (https://real-statistics.com/free-download/real-statistics-resource-pack/ (accessed on 2 July 2021) without data scaling or normalization. Spearman’s rank correlation coefficients were calculated using Excel’s built-in functions, and their significance of correlations were calculated according to standard methods [83]. Least regression lines and R^2^ values were also calculated using ordinary least squares (OLS) methods using Excel’s built-in OLS function.

## 3. Results

I analysed genome and proteome statistics for all fungal and oomycete organisms for which data were available, and I examined the correlation of these statistics —along with intrinsic proteome disorder—with growth temperatures. There is only a weak relationship between genome and proteome size and growth temperatures, but a stronger correlation between diverse measures of proteome intrinsic disorder and growth temperatures.

### 3.1. Lack of Relationship of Genome Properties with Growth Temperature in Eukaryotes

**Figure 2:**
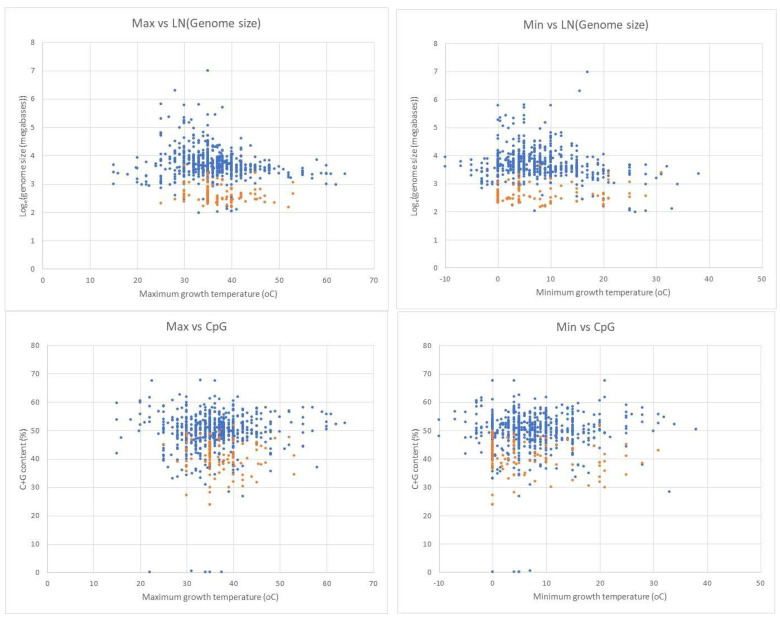
Genome properties are not correlated with maximum or minimum growth temperature. Top row; the genome size (log scale, megabases) is plotted (X axis) vs. the maximum (**left** panel) and minimum (**right** panel) growth temperature for fungi and oomycetes. Bottom row; the fraction of the genome that is G + C is plotted (X axis) vs. the maximum (**left** panel) and minimum (**right** panel) growth temperature for fungi and oomycetes. Amber coloured points are for the Saccharomycotina (discussed below). The apparent vertical columns of dots are an artefact of how growth temperature limits are often reported, which are rounded to the nearest 5 °C.

Several features of bacterial and archaeal genomes have been shown to be correlated with thermophily and hyperthermophily. Genome size is correlated with maximum growth temperature in hyperthermophilic prokaryotes [84,85,86], as is G + C content [75,87,88]. However, these are comparisons between hyperthermophiles (organisms growing above 60 °C [89,90]) and other organisms. As might be expected for a group of organisms with optimum growth temperatures of 60 °C or below, there is limited support for correlation with genome size and no support for correlation with G + C content in the dataset in this paper (Figure 2).

There is a weak negative correlation (correlation coefficient = −0.132) between genome size and maximum growth temperature, primarily because the largest fungal genomes all occur in mesophilic fungi. There is a bias for smaller genomes for both low and high maximum growth temperatures (Table 1).

The results in Table 1 suggest that large genomes are a specialization relating to an ecological niche rather than to temperature per se. A similar pattern is seen in plant groups. Plant groups with large genomes have fewer species and more restricted ecological ranges, reflecting a specific adaptative strategy [91]. I suggest that this is consistent with the hypothesis that genome size is more related to lifestyle than temperature [92,93].

It has been argued that hyperthermophiles evolve smaller genomes because the rate of chemical damage to DNA, which increases exponentially with temperature, would require an unsustainable level of mutation or DNA repair investment for a large, hyperthermophilic eukaryotic-sized genome [89,94]. While this may be true for organisms living at 120 °C, there is no support for this as a mechanism that limits eukaryotes to 60 °C.

### 3.2. Genome Sizes and Phylogenetic Classes

**Figure 3:**
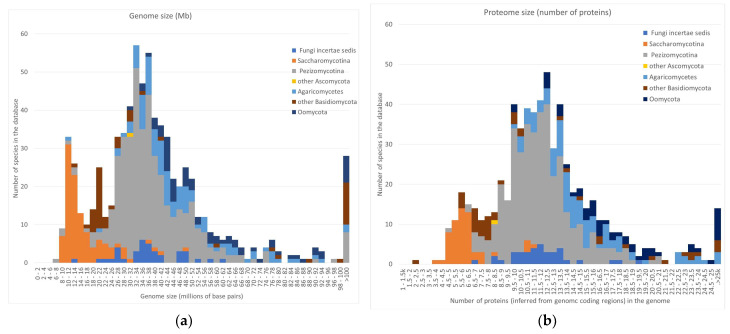
Genome and proteome sizes by taxonomic group. Distribution of genome and proteome sizes in different fungal taxonomic groups. (**a**) number of species (Y axis) vs. genome size in megabases in 2 MB bins (X axis). Major taxonomic groups considered in this study are coloured according to the legend on the right of the chart. Note that the Saccharomycotina (orange) have a different distribution from all other groups. (**b**) number of species (Y axis) plotted vs. proteome size as measured by the number of proteins in 500 protein bins. Colours are the same as in (**a**). Note again that the Saccharomycotina have a notably different distribution from all other groups.

Before exploring genome and proteome properties further, it is worth noting that the Saccharomycotina (budding ascomycete yeasts) have substantially different genome and proteome sizes compared to other fungal groups (Figure 3).

**Figure 4:**
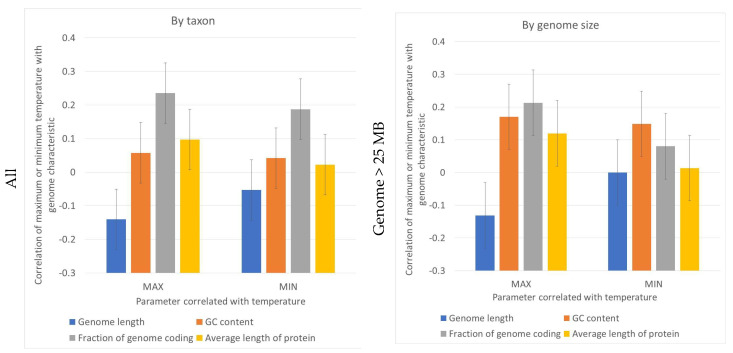

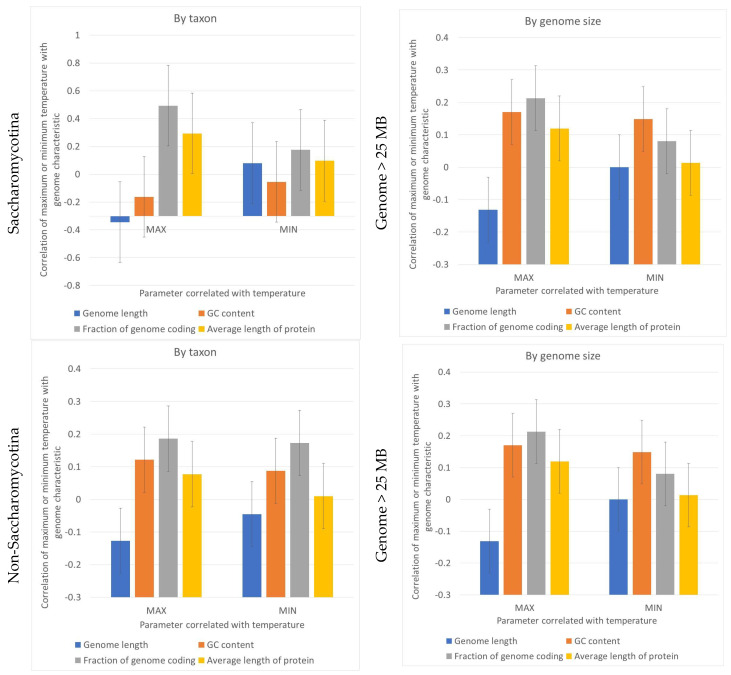
Genome coding capacity and growth temperatures. Summary statistics on correlation between fungal genome and proteome statistics and maximum growth temperature (MAX) and minimum growth temperature (MIN). Genome length is in megabases. (Note that oomycetes have been omitted from this analysis). Fraction of the genome coding is the total number of coded amino acids divided by the total genome length. Average length of protein is the total number of amino acids divided by the number of proteins. Left column pair: data for the set partitioned into all species analysed, Saccharomycotina, and non-Saccharomycotina. Right column pair: data partitioned into species with genome sizes ≤ 25 MB and >25 MB. Error bars are 95% confidence limits on the correlation given the number of species in each set.

In subsequent analyses, the Saccharomycotina were separated out to probe the relevance of the difference in genome and proteome characteristics compared to all other fungi. The number of Saccharomycotina is small (49 out of 523 proteomes); pooled analysis of all fungi did not show any different trends from those discussed below, although statistical significance was sometimes slightly lower (e.g., Figure 4). It is well known that the Saccharomycotina show reduced genomes and reduced metabolic capabilities compared to other fungi [95,96,97,98], a point further discussed below.

### 3.3. Genome Compactness and Coding Capacity Is Correlated with Maximum Temperature

Measures of gross genome size may be misleading as variation in genome size and in the degree of aneuploidy is common among fungi, especially the Saccharomycotina [99]. Aneuploidy found in wild isolates is often compensated by differences in protein turnover [100], so raw genome size may not be that important. However, genome coding capacity is seen to be significantly positively correlated with maximum and minimum temperature (Figure 4). This is a consequence on more proteins, not larger proteins, as the average size of proteins is only marginally associated with maximum and minimum temperature.

Figure 4 suggests that, for species other than the Saccharomycotina (which possess unusually small and compact genomes), genomes are more compact in species with higher maximum and, to a lesser degree, higher minimum temperatures.

There could be many explanations for these effects, including different abundances of ‘parasitic’ DNA species such as retrotransposons [101], varying sizes of centromeres and other highly repeated structures, or differences in the amount of non-coding DNA that is related to gene control. To test whether the last of these is at least a component of the relationship among genome size, genome coding density, and growth temperature limits, I explored the relationship of another core component of eukaryotic control systems: the nature of intrinsically disordered proteins in the proteome.

### 3.4. Intrinsically Disordered Fraction of Proteome Is Related to Genome Size

Intrinsically disordered proteins (IDPs) do not have a defined 3D structure in isolation but rather have extensive stretches of sequence that are disordered in solution and in crystals. As outlined in Section 1.1, IDPs have become recognized as key players in eukaryotic biology.

I calculated the predicted degree of disorder for each protein in the proteomes of the organisms studied here. The IUPred algorithm predicts a disorder score for each amino acid based on its sequence context, allowing calculation of the fraction of each protein that is likely to be disordered. This can be used to generate aggregate disorder scores for the whole proteome.

The disorder metrics used are summarized in Table 2.

**Figure 5:**
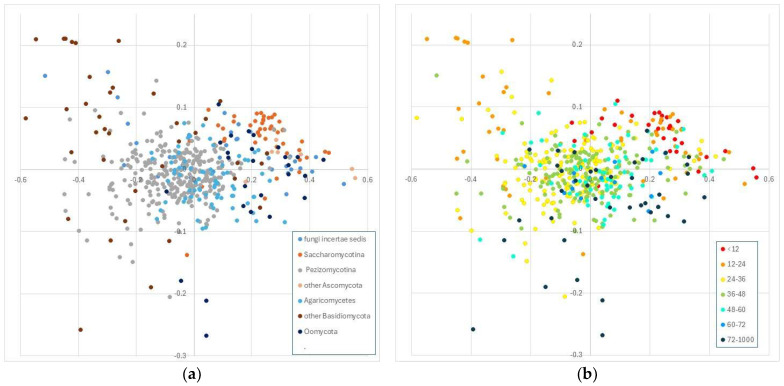
First and second principal components of aggregate proteome intrinsic disorder. Graph of the first two principal components of the dataset listed in Table 2. (**a**) points are coloured by taxonomic group. The same groups and colours are used here as in Figure 3. (**b**) points are coloured by genome size in 12 MB bins.

A principal component analysis of the parameters in Table 2 is shown in Figure 5. Adding, removing, or merging parameters from Table 2 results in broadly similar results.

None of the parameters in Table 2 include any measure of genome size, either explicitly or implicitly (for example, by counting the number of proteins or total protein length), and none of the parameters in Table 2 are significantly correlated with genome size in the overall dataset. The PCA confirms that there is no clear relationship between proteome disorder and taxon or genome size except for the smallest category of genome, the majority of which are Saccharomycotina (which, as noted above, have unusually small genomes for fungi). This suggests that the proteomes of the Saccharomycotina are anomalous compared to other fungi. Two basidiomycetes and three oomycetes with extremely large genomes are also atypical.

### 3.5. Intrinsically Disordered Protein Properties Are Correlated with Maximum Growth Temperature

In contrast to genome size, several measures of intrinsic proteome disorder are correlated with maximum growth temperature (Table 2). In the previous section, I showed that the Saccharomycotina differed significantly from other fungi in terms of the relationship between proteome disorder and genome size. Therefore, in this section, I analyse Saccharomycotina and other fungi separately. I consider the oomycetes in Section 3.6.

**Figure 6:**
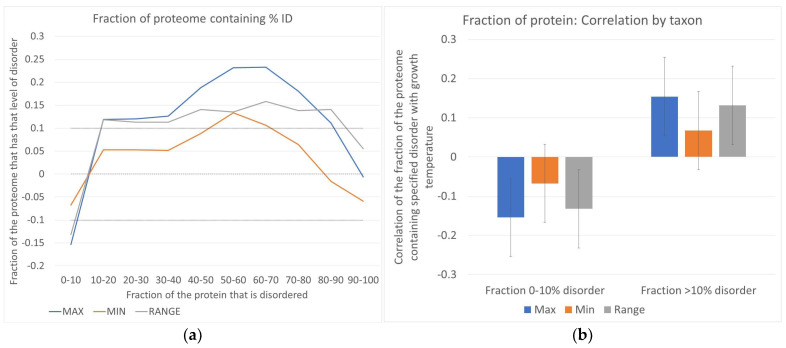
Intrinsic disorder statistic correlations with temperatures. Measures of intrinsic disorder across proteomes are significantly correlated for fungal species with genomes of >12 MB. (**a**) plot of the correlation with maximum (blue) and minimum (amber) growth temperature, and temperature range of the fraction of the proteome that is made of proteins with a percentage of disorder (X axis). Dotted lines = 95% confidence limits in correlation. (**b**) correlation of the relative number of runs of disordered amino acids across the whole proteome, for runs of >10 but <50 amino acids, between 50 and 100 amino acids, and >100 disordered amino acids with the maximum and minimum growth temperature and the range of growth temperatures. Error bars are 95% confidence limits on correlation.

Figure 6 summarizes the analysis of the correlation of two measures of intrinsic disorder in proteomes with growth temperature averages and ranges in all fungi other than the Saccharomycotina.

Figure 6a shows the correlation between the fraction of the proteome that contains a given percentage of disordered amino acids in a species and that species’ growth temperature range. The fraction of the proteome that has 10% or less disorder is the set of ‘structured’ proteins with a well-defined structure and very few amino acids that are flexible or unstructured, which are the majority of proteins. The fraction of the proteome that is composed of such well-structured proteins is significantly negatively correlated with all three measures of growth temperature: maximum, minimum, and range. The fraction that has between 10% and 90% disorder are positively correlated. There are very few 100% disordered proteins in these proteomes, so the correlation (or lack thereof) in the 100% disorder bin is not significant.

The correlations in Figure 6a imply that a higher maximum and higher minimum temperature is correlated with a higher fraction of disordered proteins, but also that the range of temperatures is independently correlated. If a high percentage of disorder was associated with high maximum and minimum temperature to the same degree, then the range would be the same for all organisms, and there would be no correlation with range.

Figure 6b shows a related statistic. In principle, Figure 6a could be due to longer stretches of disordered amino acids or a larger number of stretches of amino acids of the same length. Figure 6b shows that, at least to some extent, the correlation is due to longer stretches of disordered amino acids. I counted the number of long stretches of disordered amino acids (stretches of 100 contiguous disordered amino acids or more). The number of such long stretches of disordered residues in the whole proteome is significantly correlated with maximum and minimum growth temperature and with the range of temperatures. Again, if maximum and minimum had the same proportionality to IDP occurrence, then the range would be expected to have no significant correlation, as the maximum and minimum temperatures would track each other.

### 3.6. Oomycota Show Different Correlations Between Intrinsic Disorder and Growth Temperature

**Figure 7:**
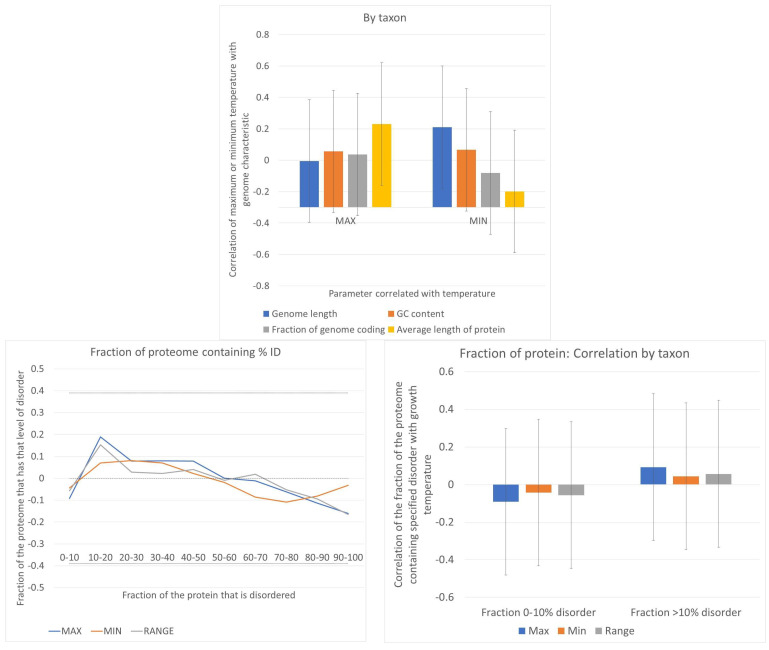
IDP statistics for oomycota. Correlation statistics are calculated as described for Figure 4 and Figure 6 above.

As the Saccharomycotina are demonstrably different from other fungi, I probed whether the results above were specific to non-yeast fungi or could be generalized to other eukaryotes. The oomycetes form a useful comparison outgroup for such a comparison. I show the results of separately analysing the oomycota in Figure 7.

Figure 7 shows the statistics for just the oomycota (of any genome size) equivalent to Figure 4 and Figure 6. As there are only 27 oomycota with proteome data in the data set, statistical significance cannot be claimed for any of these patterns. However, it is notable that the figures look quite different from fungi data in Figure 4 and Figure 6, which suggests that these conclusions may not generalize outside the Opisthokonts. Exploration of a much larger set of oomycetes may probe this further.

### 3.7. Growth Temperature Range Is Not a Function of Common Descent

**Figure 8:**
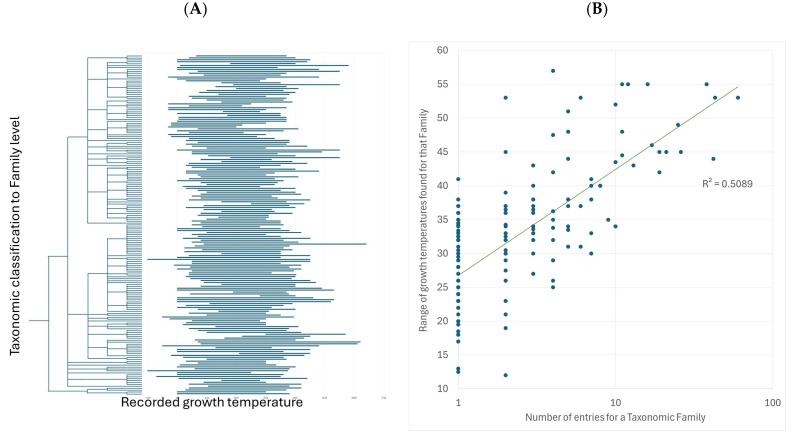
Growth temperature characteristics of fungi by taxonomic group. (**A**): taxonomic classification of the fungi used in this study: levels of classification are Kingdom (Fungi or SAR), Phylum, Class, Order, Family. Y axis: classification tree. X axis: range of growth temperatures from minimum to maximum found in that Family. (**B**): Range of growth temperatures within a Family (Y axis) plotted vs. number of species-level entries in the dataset within that family (X axis). Green line is ordinary least squares match for log(X) vs. Y.

A possible explanation for the correlations observed above would be that some groups of fungi have adapted to grow at high or low temperatures, and have also inherited specific genome properties, and so the association of genome properties with growth temperature is an artefact of common descent. This can be tested experimentally as discussed below, but the likelihood of a phylogenetic explanation can be probed with the observational dataset. In Figure 8, I show two tests of this hypothesis by grouping the fungi in this dataset into Families (one major taxonomic level above Genus), and quantifying the reported range of temperatures in each Family.

Figure 8A shows there is no obvious trend between taxonomy and growth temperature, which suggests that common descent cannot explain the relationship between genome properties and growth temperature.

An alternative explanation for the patterns in Figure 8A is shown in Figure 8B, which plots the observed range of growth temperatures within a Family against the number of species in that Family for which growth temperatures have been measured. There is a clear trend (R^2^ = 0.5089) between the intensity of publication (and therefore, plausibly, of experimental study) of a Family and the range of growth temperatures seen in members of that Family. I return to this point in the discussion below.

### 3.8. High Growth Temperature Is Associated with Large Range of Growth Temperatures

**Figure 9:**
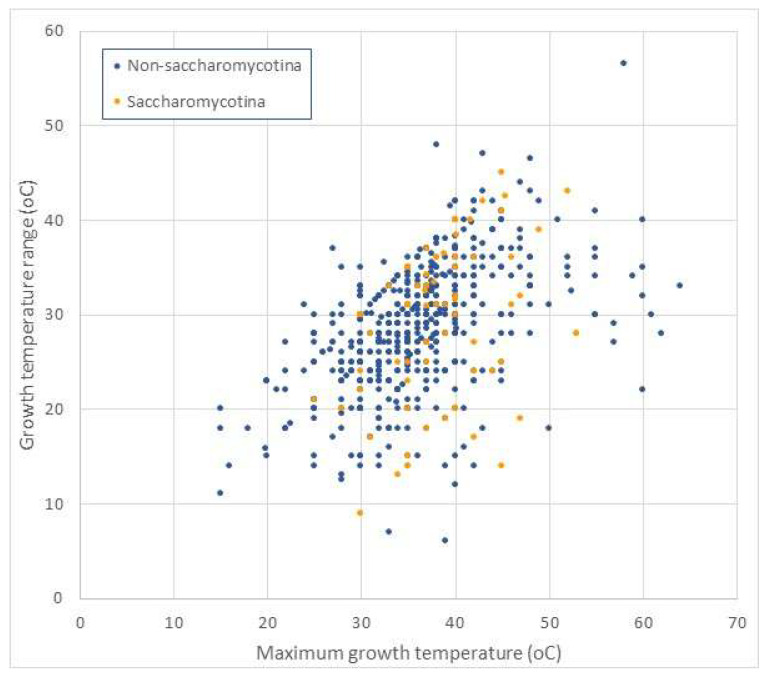
Maximum growth temperature vs. growth temperature range.

It is not clear why a higher content of IDPs, both in terms of fraction of disorder and length of disordered regions in proteins, should be correlated with maximum and minimum growth temperature and with the range of growth temperatures. Adaptation to non-mesophilic temperature is an adaptation like any other and will come with no more demands on cellular control processes than adaptation to metabolic substrates or infectious potential in pathogens. However, temperature range is itself correlated with maximum temperature, as shown in Figure 9.

Figure 9 shows a correlation (correlation coefficient 0.449) between the maximum temperature at which a fungus can grow and the range of temperatures over which that fungus can grow. This applies more weakly to Saccharomycotina (significance of a correlation of 0.241 in Saccharomycotina *p* = 0.013) than for the other organisms (correlation 0.507), again pointing to the Saccharomycotina as outliers.

I therefore hypothesize that organisms adapting to high temperatures must also adapt to a wider range of temperatures. The reasons for this, and the thermodynamic implications, are discussed in Section 4.

### 3.9. Limitations of This Study

The principal limitation in the dataset used in this study is in the minimum growth temperature, and hence, in the range of growth temperatures. Defining the temperature at which a fungus cannot grow depends on the definition of “cannot grow”. The growth rate of many fungi slows with decreasing temperature, but some growth can be seen at low temperatures, depending on how growth is measured and the timescale over which growth is examined. As it is rare for experimenters to give these details, comparison of stated minimum growth temperatures between studies is difficult. Additionally, studies on psychrophiles often focus on freezing, dehydration, or UV tolerance rather than minimum growth temperature [102]. The minimum growth temperature of psychrophiles is often given as the freezing point of the medium on which they grow (natural or artificial); however, fungi have been measured to metabolise and grow at −35 °C in a specially constituted medium that remains liquid at that temperature [103], suggesting that observed minima near the freezing point of water may be set by that freezing point and not by the biochemistry of the cell.

By contrast, maximum growth temperature can be ascertained to within a few degrees, as the maximum is often only a few degrees above the optimum growth temperature. The gradient on the curve of growth rate vs. temperature is very steep on the high temperature end, with the difference between tolerance and lethality often being only a few degrees for a wide range of animal species (100%/degree), even though a number of metabolic processes only increase by 7%/degree [104]. See [80] for more discussion on determining maximum and minimum growth temperatures.

I also note that fungi are grown under very different conditions in different studies, which may affect low or high temperature limits. This is in part unavoidable, as the growth conditions for different organisms are different, so it is not possible to grow a fungus that is a wood-degrading saprophyte under the same conditions as an obligate mammalian parasite. In principle, however, an experimental programme to probe growth rates under a small set of defined conditions and with a consistent definition of what constitutes growth could address both the range and the growth condition limitations outlined here.

All three limitations mentioned above are exacerbated by the limited study of fungi. Figure 8 illustrates that the more members of a Family of fungi that are studied, the wider the range of growth temperatures found for that Family. This hints that many more cold- or hot-tolerant fungi may exist to be discovered in little-studied taxonomic groups.

The further conceptual limitation to this study is that the statistics above show correlation, not causation. Any suggestion of a reason or a mechanism behind those correlations must therefore be hypothesis, which can thereby point to tests for that hypothesis.

## 4. Discussion: A Thermodynamic Limit on Maximum Growth Temperature of Eukaryotes

The results above suggest that adaptation to a range of temperatures requires increasing flexibility of control systems within the cell, illustrated by an increasing content of intrinsically disordered proteins. An organism that is adapted to high temperatures must be able to grow, and reproduce, at elevated temperatures, but those temperatures occur in an ecological context. Specifically, there are few environments where temperatures are always high (or low). Even the Antarctic valleys experience very substantial temperature ranges [105], and environments where thermophilic fungi are typically found such as compost heaps or rotting vegetation are transient.

I therefore hypothesize that adaptation to temperatures substantially different from the mesophilic average (at an ecosystem’s latitude) necessitates adaptation to active survival in mesophilic temperatures as well as at elevated temperature. This may apply to a single organism or to a multi-generational lineage over any timescale shorter than that needed to evolve a new temperature tolerance. It is well known that ectothermic organisms that are adapted to more thermally stable environments have narrower thermal tolerances than ones adapted to environments where the temperature fluctuates substantially (e.g., [106,107,108,109,110,111]). This work suggests the same is true globally of fungi (Figure 9); I hypothesize that the correlations between genome and proteome structure and maximum temperature observed here arise because specific adaptive programmes are needed for organisms that can adapt to grow over a wide range of temperatures. Higher growth temperature requires adaptability to larger temperature range, which in turn requires more flexibility in the control architecture of the cell as implemented in (among other things) IDPs.

However, the functions of IPDs are inherently entropy-dominated, and hence, temperature-sensitive. I hypothesize that the thermodynamics of structural control, and specifically of IDP function, in fungi is an explanation for the observed limit of growth of fungi to 65 °C.

The thermodynamics of large-scale structural changes are dominated by entropic changes—that is, the change in order of both macromolecules and solvent on assembling or disassembling the structures [112,113]. This applies to IDP function, but also to all aspects of eukaryotic gene control, which is strongly dependent on large-scale order in the nucleus. The binding affinity of a single protein to DNA is sometimes dominated by the enthalpy of binding; sometimes it is a balance between enthalpy and almost equally large entropy effects, depending on the protein and gene concerned (see for example [114,115,116,117,118,119] and refs therein). The thermodynamics of the reactions that drive the structural organization of DNA in eukaryotes, including supercoiling [120], binding of DNA to the nucleosome [121,122,123], and looping of DNA into large higher order structures [123,124], are dominated by large entropy changes [112,113]; many of these processes involve IDPs. The enthalpy changes in these reactions are only just sufficient to counter the adverse entropy changes, so these reactions have a small net free energy change [122,123,124], as they must if they are to be easily reversible to allow gene control without expenditure of enormous energy. It is plausible, although it has not been tested, that similar entropy changes accompany the formation of large control structures outside the nucleus.

All processes in biochemistry proceed in a forward direction because they are thermodynamically favoured and kinetically enabled. The Gibbs free energy of a process (the free energy under constant temperature and pressure, but potentially variable volume) is given by

ΔG = ΔH − TΔS(1)

where ΔG is the Gibbs free energy change of the reaction, ΔH is the enthalpy change, ΔS is the entropy change, and T is the absolute temperature. If ΔG is less than zero, then the reaction progresses with a loss of free energy and can therefore proceed spontaneously. The entropy-dominated, IDP-based regulation systems in eukaryotes are therefore expected to be sensitive to temperature; their large ΔS means that a small change in T will result in a large change in ΔG, and so a large change in ΔH would be needed to compensate for a change in TΔS following a temperature change. Changes in the interaction energies of proteins can be effected by changing the protein’s sequence or by changing post-translational modifications, including protein abundance. It is plausible that there is a limit to the degree to which ΔH can be adapted without major sequence change to the protein. Sequence changes can occur over evolutionary time, but not within the lifetime of an individual organism. Thus, an organism is limited to the degree that it can adapt the ΔH of a process to compensate for a change in TΔS, i.e., to compensate for a change in temperature. This limit appears functionally to be in the range of 30 °C to 40 °C from Figure 9. For prokaryotes, TΔS is much smaller because of their smaller reliance on entropy-driven processes, and so the range over which their biochemistry could function is much larger.

The observations of IDP function, observed temperature range, and the thermodynamics of IDP and gene control functions imply that the limit of eukaryotic life to 65 °C or below is a result of the average temperature of the planet, and not aspects of the chemical or cellular structure of eukaryotes. Under this formulation, there is no reason why eukaryotic life should not function at 80 °C, providing that its environment was static and did not even occasionally require that life to function at 20 °C.

Dallinger constructed such an environment. He isolated four samples of a complete pond micro-ecosystem from an English pond, including phototrophs, sealed them into glass bottles, and then incubated them under lights in a water bath. He gradually raised the temperature of these closed ecosystems from 20 °C over a period of 7 years [125], observing the organism through a low-power microscope to confirm that there were still living organisms in the samples. Frequently rising temperature led to substantial changes in population morphology suggestive of stress; when this happened, Dallinger paused the temperature increase until the morphology of the surviving organisms appeared more typical. Because the temperature did not fluctuate, organisms could adapt to a constant temperature, and he succeeded in creating a pond ecosystem that grew at 70 °C before the apparatus broke (why and how it broke was not specified). This confirms that 65 °C is not an absolute limit set by eukaryotic biology, but an ecological limit that can be broken by creating a suitable permissive environment.

### Not All Complex Systems Show Entropically Driven Temperature Dependence

**Figure 10:**
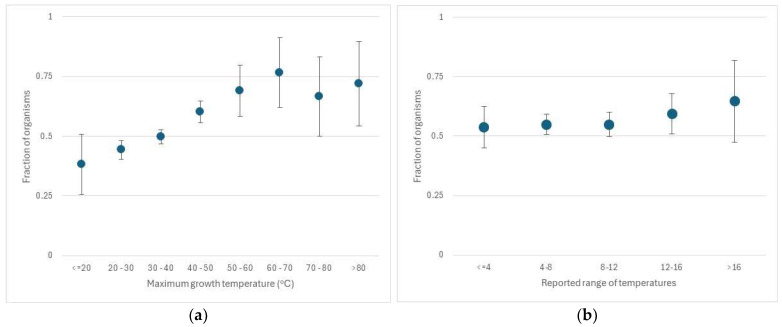
Fraction of organisms reported to be motile as a function of their optimal growth temperature. Fraction of organisms reported in the International Journal of Systematic and Evolutionary Microbiology that are motile, as a function of growth temperature (where that is stated–2629 organisms) or range of temperature where a temperature range is stated (1215 organisms). Error bars are √(N)/N, where N is the number of organisms in each temperature range. (**a**) Fraction of organisms reported to be motile plotted as a function of maximum growth temperature for those organisms. (**b**) Fraction of organisms reported to be motile platted as a function of the reported range of growth temperatures of those organisms.

The argument stated above also implies that complex cellular systems that are primarily enthalpy driven, not entropy driven, should have much wider temperature ranges than entropy-dominated systems. I tested this using the bacterial flagellal motor as an example. The bacterial flagellal motor is a multiprotein complex with dynamic movement [126], but its mechanism involves relatively small changes in protein structure and is unidirectional [127]; therefore, I hypothesise that its thermodynamics would be less dominated by entropic changes. As the hypothesis above predicts, the abundance of motility is not obviously related to growth temperature or the range of growth temperatures for bacterial species (Figure 10).

The assembly of other complex cellular structures specific to the eukaryotic cell is also driven by entropy. Actin polymerisation to form microfilaments (“skeletal” structures within the eukaryotic cell that are central to many cell functions) is driven by a balance between large entropic changes and equally large, balancing enthalpic changes. The enthalpy changes have been studied in eukaryotes adapted to different temperatures between 0 °C and 40 °C [128]. Each actin is specialized to have an enthalpy of polymerisation that balances the entropy lost upon assembly of these complex multiprotein systems at the organism’s optimal temperature, and as a result, they do not ‘work’ at higher or lower temperatures. However, the presence of actin-like proteins in archaea, including thermophilic archaea, suggests that this mechanism is more readily adaptable to high temperature [129].

## 5. Discussion and Implications

I have presented statistical evidence that the maximum growth temperature of fungi is correlated with the range of temperatures over which they can grow, and that the minimum, maximum, and range of growth temperatures of fungi are correlated with the genome content of intrinsically disordered proteins. I have suggested the hypothesis that thermodynamics can, at least in part, explain these effects because processes with a large change in entropy, and especially ones involving intrinsically disordered proteins, have a limit to the degree to which they can be adapted to operate at different temperatures. However, this would not be an absolute limit but rather one set by evolutionary contingency and the realities of temperature ranges seen in real-world ecologies. In principle, a eukaryote could be evolved in a laboratory to grow at at least 70 °C, providing the temperature was constant, and one Victorian experiment hints that this is indeed possible [125].

### 5.1. Experimental Tests of the Hypothesis

I suggest two sets of experimental verifications of this hypothesis.

The first is to measure directly the contributions of entropy and enthalpy to gene control mechanisms, and their adaptation to temperature, in vivo. Experimental thermodynamic analysis has been performed on the interaction between DNA and proteins in prokaryotic systems in vitro [112,116,118,119] and on the interaction between DNA, nucleosomes, and higher chromatin structure in eukaryotic systems in vitro [114,115,121,123,124]. The thermodynamics of IDPs’ role in phase separation phenomena has also been studied. These studies are impractical to replicate directly in vivo, but a variation of the thermal shift assay that probes protein stability as a function of temperature [130,131] may enable the thermodynamics of IDPs to be inferred in their native context by comparison of their structural stability as a function of temperature in the presence or absence of binding partners. In addition, recent AI-based modelling techniques may have the power to model IDP complexes in a realistic in vivo environment [132].

However, a simpler test is directly suggested by the hypothesis outlined here. If this hypothesis is correct, then a population of a eukaryote grown in an environment where the temperature was static would be free to adapt to any arbitrary temperature. This was the experiment reported by [125], as summarized above. Despite the substantial literature on experimental evolution studies (reviewed in [133,134]), there have been few attempts to test the limits of eukaryotic thermal adaptation, although several have been performed on *E. coli* [135,136,137].

A replication of [125] with modern analytical tools would allow genome sequencing to show what aspects of the organism’s proteome had changed to allow it to grow at an elevated temperature. Systematic studies of mutations already conducted would provide the basis for understanding the role of different mutations in different protein types (e.g., [138,139,140]). This would be a long-term experiment, but perhaps not as long-term as [125]; a relatively short duration of selection can significantly increase the maximum growth temperature of filamentous fungi [141].

### 5.2. Are the Budding Yeasts Good Models for Temperature Adaptation?

The experimental sketch above raises the question of which species to attempt to adapt. I suggest that the species selected should not be a yeast. A sidenote from this study is that the Saccharomycotina (Budding Yeasts) appear to have quite different statistics of genome and IDP content with respect to temperature than all other fungi. They also have much smaller genomes, the result of genome reduction from the Budding Yeast Common Ancestor [95,96], including the loss of metabolic capabilities relating to biomass breakdown, such as secreted hydrolases and secondary metabolite biosynthesis; they also show selection for mitochondrial-related genes [97,98]. This metabolic narrowing and optimization of bioenergetic metabolism is likely an adaptation to their ecological niche of transient nutrient-rich environments such as fruit, nectars, and leaf surfaces [142,143] and very short life cycles under optimal conditions. This extreme r-strategy may be a driver behind both genome reduction and the loss of many of the more complex control mechanisms seen in other fungi. Small genome size has made them attractive as models for many studies but atypical of other fungi, and possibly of other Opisthokonts. For temperature adaptation studies, I suggest Budding Yeasts may be poor models. It remains to be seen whether they are also poor models for other in vitro evolution experiments that require complex changes of the genetic programme (e.g., [144,145]).

## 6. Conclusions

No eukaryotes can grow and complete a life cycle at above 65 °C. I present arguments that this is not a result of specifics of chemistry but of the thermodynamic implications of the widespread use of entropy-driven processes in cellular control, and specifically the role of intrinsically disordered proteins, coupled with the range of temperatures that any organisms will have to adjust to in a real-world ecology. This implies that eukaryotic terrestrial life could adapt to higher temperatures if provided with a stable temperature environment over the long term. It also implies that 65 °C is not necessarily a limit for complex life to evolve in environments different from those on the surface of Earth today.

## Figures and Tables

**Table 1:** Large fungal genomes are associated with mesophilic maximum growth temperatures ^1^.

Genome Size	Temp < 30 °C	30 °C ≤ Temp ≤ 40 °C	Temp > 40 °C
Genome ≤ 40 MB	42	324	118
Genome > 40 MB	37	180	18

^1^ Number of fungi and oomycetes in the dataset that have genomes smaller or larger than 40 megabases (rows) and maximum growth temperatures below 30 °C, above 40 °C, or between those temperatures (columns). The distribution is significantly different from random (Chi^2^ = 40.66, *p* << 0.001).

**Table 2:** Proteome intrinsic disorder statistics used in principal component analysis (PCA).

Measure		Comment	Correlation with Genome Size ^1^	Correlation with Maximum Growth Temperature ^1^
average of (average disorder/protein)		Average across the proteome of A, where A is the number of disordered amino acids in a protein divided by its length	−0.004	0.046
Average length of maximum run		Average length of the longest run of disordered amino acids in each protein	−0.111	0.152
Fraction of proteins with X-Y% disorder	0–10%	Average for the whole proteome D, where D is the number of disordered amino acids in a protein divided by the length of that protein	0.037	−0.102
	10–20%		−0.055	0.095
	20–30%		−0.037	0.078
	30–40%		−0.037	0.073
	40–50%		−0.044	0.122
	50–60%		−0.045	0.152
	60–70%		−0.019	0.150
	70–80%		0.037	0.111
	80–90%		0.065	0.052
	90–100%		0.102	−0.040
Relative number of runs of X-Y disorder	10–19	Number of runs of disordered amino acids of length L in the whole proteome divided by the number of proteins in the proteome	−0.112	0.039
	20–29		−0.119	0.054
	30–39		−0.120	0.076
	40–49		−0.118	0.086
	50–59		−0.105	0.101
	60–69		−0.129	0.123
	70–79		−0.121	0.123
	80–89		−0.128	0.136
	90–99		−0.117	0.139
	≥100		−0.117	0.178

^1^ Values in bold are significantly different from a null hypothesis of no correlation at *p* ≤ 0.05 level Bonferroni corrected for multiple testing.

## Data Availability

The original contributions presented in the study are included in the article/Supplementary Material, further inquiries can be directed to the corresponding author.

## Supplementary Materials

The following supporting information can be downloaded at: https://www.mdpi.com/article/10.3390/life16061016/s1, Database table of fungal species, genome and proteome characteristics and growth temperatures used in this study.
